# Risk factors of short-term mortality after acute nonvariceal upper gastrointestinal bleeding in patients on dialysis: a population-based study

**DOI:** 10.1186/1471-2369-14-97

**Published:** 2013-04-26

**Authors:** Ju-Yeh Yang, Tsung-Chun Lee, Maria E Montez-Rath, Glenn M Chertow, Wolfgang C Winkelmayer

**Affiliations:** 1 Division of Nephrology, Department of Medicine, Stanford University School of Medicine, 1070 Arastradero Rd., Suite 313, Palo Alto, CA, 94304, USA; 2 Division of Nephrology and Department of Internal Medicine, Far Eastern Memorial Hospital, No.21, Sec. 2, Nanya S. Rd., Banciao Dist., New Taipei City, 220, Taiwan; 3 Division of Gastroenterology, Department of Internal Medicine, National Taiwan University Hospital and National Taiwan University College of Medicine, No.7, Chung Shan S. Rd. (Zhongshan S. Rd.), Zhongzheng Dist., Taipei City, 10002, Taiwan

**Keywords:** Gastrointestinal hemorrhage, Risk factors, Mortality, Dialysis

## Abstract

**Background:**

Impaired kidney function is an established predictor of mortality after acute nonvariceal upper gastrointestinal bleeding (ANVUGIB); however, which factors are associated with mortality after ANVUGIB among patients undergoing dialysis is unknown. We examined the associations among demographic characteristics, dialysis-specific features, and comorbid conditions with short-term mortality after ANVUGIB among patients on dialysis.

**Methods:**

Design: Retrospective cohort study. Setting: United States Renal Data System (USRDS), a nation-wide registry of patients with end-stage renal disease. Participants: All ANVUGIB episodes identified by validated algorithms in Medicare-covered patients between 2003 and 2007. Measurements: Demographic characteristics and comorbid conditions from 1 year of billing claims prior to each bleeding event. We used logistic regression extended with generalized estimating equations methods to model the associations among risk factors and 30-day mortality following ANVUGIB events.

**Results:**

From 2003 to 2007, we identified 40,016 eligible patients with 50,497 episodes of ANVUGIB. Overall 30-day mortality was 10.7% (95% CI: 10.4-11.0). Older age, white race, longer dialysis vintage, peritoneal dialysis (vs. hemodialysis), and hospitalized (*vs.* outpatient) episodes were independently associated with a higher risk of 30-day mortality. Most but not all comorbid conditions were associated with death after ANVUGIB. The joint ability of all factors captured to discriminate mortality was modest (*c*=0.68).

**Conclusions:**

We identified a profile of risk factors for 30-day mortality after ANVUGIB among patients on dialysis that was distinct from what had been reported in non-dialysis populations. Specifically, peritoneal dialysis and more years since initiation of dialysis were independently associated with short-term death after ANVUGIB.

## Background

Acute nonvariceal upper gastrointestinal bleeding (ANVUGIB) is common among patients on dialysis and associated with poor outcomes. Using a stringent criterion to ascertain ANVUGIB events from Medicare claims, the occurrence rate of ANVUGIB among patients on dialysis was estimated at 57 episodes per 1000 person-years [1], almost two orders of magnitude higher than in the general population. Approximately 12% of patients died within 30 days after the event [1]. Wasse et al. have previously analyzed the United States Renal Data System (USRDS) to identify risk factors for incident upper gastrointestinal bleeding (UGIB) among patients with end-stage renal disease (ESRD) [2]. However, there are no reports on the risk factors for outcomes after UGIB in this population.

Although ANVUGIB is a potentially life-threatening condition, most patients in the general population are at low risk for mortality and often die from other complications, rather than from bleeding itself [3]. Determinants of mortality following ANVUGIB in the general population included age, certain comorbidities (including cardiovascular disease, liver disease, kidney function, and malignancy), specific endoscopic findings, and biometric and laboratory data at presentation (such as blood pressure and hemoglobin concentrations) [4-7]. We conducted the present study to establish predictors of 30-day mortality after ANVUGIB in a national cohort of U.S. patients undergoing dialysis. While we expected several established risk factors for mortality on dialysis to also be associated with short-term mortality after ANVUGIB, we focused on identifying risk factors that are of particular pertinence to the bleeding event, such as history of earlier ANVUGIB episodes, whether the bleeding was peptic ulcer-related, as well as the care setting of its diagnosis and the most recent dialysis modality before the event. We also hypothesized that risk factors might be modified by age, sex, race, and dialysis modality.

## Methods

### Data source

We used the USRDS database for this study. The USRDS contains health care claims submitted by providers to Medicare on most patients with ESRD receiving maintenance dialysis care or kidney transplantation in the U.S. Most patients with ESRD qualify for Medicare coverage independent of their age, which enables research on a large number of subjects younger than 65 years, a population for whom Medicare claims are usually not available.

### Study population

We analyzed all patients who underwent maintenance dialysis in the US between January 1, 2003 and December 31, 2007. Only those patients continuously covered by traditional fee-for-service Medicare at least for one year before the ANVUGIB episode were included to ensure adequate and uniform ascertainment of comorbidities. Patients under the age of 18 years or whose kidney function recovered were excluded.

### Identification of ANVUGIB episodes

From claims collected during periods of Medicare eligibility only, we identified ANVUGIB events using a modified stringent criterion proposed by Targownik et al. [8]. As described in recent work [1], ANVUGIB events were defined from a group of ICD-9 diagnostic codes, which specify the identified cause of the bleeding, such as gastroesophageal laceration-hemorrhage (530.7) or gastric ulcer with hemorrhage (531.0). Additionally we included several important lesion-specific events not considered by Targownik et al. [8] (e.g., 537.83 for angiodysplasia of stomach and duodenum with hemorrhage; 537.84 for Dieulafoy lesion (hemorrhagic) of stomach and duodenum). Detailed codes are listed in Additional file 1.

To avoid lack of specificity while ascertaining ANVUGIB events from outpatient claims, we defined a single ANVUGIB event based on the presence of two outpatient claims within a 7-day time period. Alternatively, one outpatient claim was considered sufficient if it was accompanied by a claim for esophagogastroduodenoscopy performed on the same date [9]. Further, if the onset dates of two events were within a 30-day period, these two events were counted as a single episode and the earlier event was used as the index date marking the beginning of follow-up [9].

### Study outcomes

The primary outcome of interest was short-term (30-day) mortality following an episode of ANVUGIB, defined as having died within 30 days of the ANVUGIB diagnosis date regardless of discharge status [9,10].

### Candidate predictors

We obtained patient-level characteristics such as age, sex, race, vintage (time since first ESRD date), and the presumed primary kidney disease causing ESRD from the Medical Evidence Report CMS-2728. We ascertained the most recent dialysis modality (hemodialysis *vs.* peritoneal dialysis) and any history of kidney transplantation from treatment history files. We used the 60-day file constructed from the detailed file to avoid the concern that patients had been switched the modality just for the event. We ascertained a large number of comorbidities from one year of inpatient and outpatient claims preceding the ANVUGIB event using validated algorithms, where available: diabetes, heart failure, coronary artery disease, chronic obstructive pulmonary disease, cerebral vascular disease, hypertension, arrhythmia, valvular heart disease, peripheral vascular disease, chronic liver disease, and malignancy [1]. To define prior ANVUGIB history, we traced back to all available claims in our database (since 1996).

### Statistical analysis

We used logistic regression models to evaluate the associations among potential risk factors and 30-day mortality. As each subject was eligible to contribute more than one episode, correlations among observations within subject are expected. We applied generalized estimating equations (GEE) with an exchangeable working correlation structure to adjust for the expected dependence of observations within each patient [11]. Since only 0.04% and 0.35% patients had their race and dialysis modality missing, respectively, we conducted complete case analyses.

We were *a priori* interested in the interactions of age, sex, race, and dialysis modality with other risk factors. To avoid over fitting the data while exploring for interactions among covariates, we randomly split our data into a derivation (60%) and a validation sample (40%). In this exploratory phase, we fit logistic regression models assuming independence of observations where each interaction was added as a covariate to the main effects model. We applied the minimum Akaike information criterion (AIC) procedure [12], which selects the model with the smallest AIC among the set of models tested. Each model’s AIC is computed in the validation sample using parameter estimates from the derivation sample. If a significant interaction were to be found in the derivation sample, its significance would be validated by a decline in AIC value in the validation sample.

Once the final model was found, which included all covariates and validated interactions, it was fit in the full sample using GEE. We reported unadjusted and multivariable-adjusted odds ratios (OR) and 99% confidence intervals (CI) for each factor. All analyses were performed using SAS 9.2 software (The SAS Institute Inc, Cary, NC).

This work was approved by the Institutional Review Board of Stanford University School of Medicine and conducted under a data use agreement to Dr. Winkelmayer from the National Institute of Diabetes and Digestive and Kidney Diseases (NIDDK). This manuscript was reviewed by NIDDK and approved for submission.

## Results

From 2003 to 2007, we identified 50,497 eligible episodes of ANVUGIB, which were experienced by 40,016 unique patients. The number of episodes per patient ranged from 1 to 14 [10^th^ percentile: 1; 90^th^ percentile: 2]. Characteristics of the study population are shown in Table 1: the average age was 63 years and slightly more than half of the episodes were in men and in whites. The vast majority of episodes occurred in patients undergoing HD (93.6%), and the median time since first ESRD treatment was 3.8 years. All comorbidities were highly common; almost two-thirds had diabetes and almost three-quarters had been diagnosed with hypertension in the previous year. One-third of episodes (17,645) occurred in patients in whom a previous episode was recorded and 40.6% (7,164) of those previous episodes occurred before 2003. The diagnosis most commonly identifying ANVUGIB episodes was gastritis or duodenitis with hemorrhage (29.1%, Table 2).

**Table 1:** Patient characteristics at each episode, by 30-day survival status

	**All episodes**	**Survived**	**Deaths**
Number (row %)	50497	45084 (89.3)	5413 (10.7)
Age (years; ± standard deviation)	62.9 (±14.7)	62.3 (±14.8)	68.0 (±13.2)
18 to <50 years (%)	10232 (20.3)	9670 (21.4)	562 (10.4)
50 to <65 years (%)	15148 (30.0)	13775 (30.6)	1373 (25.4)
65 to <75 years (%)	13492 (26.7)	11864 (26.3)	1628 (30.1)
≥75 years (%)	11625 (23.0)	9775 (21.7)	1850 (34.2)
Male	27025 (53.5)	24092 (53.4)	2933 (54.2)
Race			
White (%)	25962 (51.4)	22770 (50.5)	3192 (59.0)
Black (%)	21771 (43.1)	19849 (44.0)	1922 (35.5)
Other (%)	2764 (5.5)	2465 (5.5)	299 (5.5)
Dialysis modality			
Hemodialysis (%)	47274 (93.6)	42277 (93.8)	4997 (92.3)
Peritoneal dialysis (%)	3223 (6.4)	2807 (6.2)	416 (7.7)
Dialysis vintage [years; median (IQR)]	3.8 (2.2; 6.2)	3.8 (2.2; 6.2)	4.0 (2.3; 6.4)
≥6 years (%)	13463 (26.7)	11959 (26.5)	1504 (27.8)
3 to <6 years (%)	17696 (35.0)	15821 (35.1)	1875 (34.6)
1 to < 3 years (%)	19338 (38.3)	17304 (38.4)	2034 (37.6)
History of comorbid conditions			
Diabetes (%)	32181 (63.7)	28625 (63.5)	3556 (65.7)
Hypertension (%)	36939 (73.2)	32925 (73.0)	4014 (74.2)
Coronary artery disease (%)	15147 (30.0)	13299 (29.5)	1848 (34.1)
Cerebral vascular disease (%)	8750 (17.3)	7538 (16.7)	1212 (22.4)
Heart failure (%)	26833 (53.1)	23437 (52.0)	3396 (62.7)
Arrhythmia (%)	20392 (40.4)	17596 (39.0)	2796 (51.7)
Valvular heart disease (%)	15379 (30.5)	13310 (29.5)	2069 (38.2)
Peripheral vascular disease (%)	18792 (37.2)	16231 (36.0)	2561 (47.3)
Chronic obstructive lung disease (%)	13927 (27.6)	11998 (26.6)	1929 (35.6)
Any cancer (%)	4335 (8.6)	3588 (8.0)	747 (13.8)
Upper gastrointestinal cancer* (%)	250 (0.5)	198 (0.4)	52 (1.0)
Chronic liver disease (%)	6423 (12.7)	5530 (12.3)	893 (16.5)
History of kidney transplant (%)	3119 (6.2)	2849 (6.3)	270 (5.0)
History of ANVUGIB (%)	17645 (34.9)	16021 (35.5)	1624(18.9)

Abbreviation: IQR – interquartile range.

* Upper gastrointestinal cancer (ICD-9 diagnosis codes 150–152).

**Table 2:** Frequencies of specific diagnoses among 50,497 ANVUGIB Episodes

	**Count**	**Proportion of episodes**
Esophageal ulcer/hemorrhage	6,370	12.6%
Gastric ulcer with hemorrhage	9,788	19.4%
Duodenal ulcer with hemorrhage	5,152	10.2%
Peptic ulcer with hemorrhage	1,166	2.3%
Gastrojejunal ulcer with hemorrhage	397	0.8%
Gastritis/duodenitis with hemorrhage	14,702	29.1%
Angiodysplasia/Dieulafoy lesion with hemorrhage	4,479	8.9%
Hematemesis	17,643	34.9%

Note: A single episode might have had more than one diagnosis associated with it.

Five thousand, four hundred and thirteen patients died within 30 days of ANVUGIB, representing 13.5% of patients and 10.7% of episodes. Table 3 lists the unadjusted and multivariable-adjusted OR and 99% CI of all independent variables considered in this study. The full model exhibited only modest discrimination with a *c* statistic of 0.68 and an R^2^ of 0.04. The 30-day death rate in the lowest decile of predicted probabilities of death was 2.3%, as compared to 21.9% in the highest decile. Older age, white race, longer dialysis vintage, peritoneal dialysis (*vs.* hemodialysis), and hospitalized (vs. outpatient) episodes were associated with higher risk of 30-day mortality after ANVUGIB. Patients with no recorded history of ANVUGIB were more likely to die within 30 days after an episode. Most comorbidities were associated with 30-day mortality after ANVUGIB except for diabetes mellitus, coronary artery disease (main effect OR: 1.04; 99% CI 0.92-1.18), and hypertension.

**Table 3:** Odds ratios for 30-day mortality after acute nonvariceal upper gastrointestinal bleeding

		**Odds ratio (99% Confidence Interval)**	
**Variable**	**Category**	**Unadjusted**	**Adjusted**
Age	18 to <50 years	1.0 (reference)	1.0 (reference)
	50 to <65 years	1.71 (1.49 to 1.95)	1.54 (1.34 to 1.77)
	65 to <75 years	2.35 (2.06 to 2.68)	2.04 (1.77 to 2.35)
	≥75 years	3.24 (2.84 to 3.69)	2.79 (2.42 to 3.21)
Sex (Males vs. females)*		1.03 (0.96 to 1.11)	
	Among patients with CAD		0.94 (0.82 to 1.07)
	Among patients without CAD		1.12 (1.02 to 1.23)
Race	White	1.0 (reference)	1.0 (reference)
	Black	0.69 (0.64 to 0.75)	0.80 (0.73 to 0.87)
	Others	0.86 (0.73 to 1.02)	0.96 (0.81 to 1.13)
Vintage	1 to <3 years	1.0 (reference)	1.0 (reference)
	3 to <6 years	1.02 (0.93 to 1.11)	1.11 (1.01 to 1.21)
	≥6 years	1.08 (0.98 to 1.18)	1.37 (1.24 to 1.52)
Dialysis modality	peritoneal vs. hemodialysis	1.25 (1.09 to 1.45)	1.54 (1.32 to 1.78)
Bleeding likely peptic ulcer related		0.99 (0.92 to 1.07)	0.93 (0.86 to 1.01)
Hospitalized bleeding episode		2.17 (1.86 to 2.54)	2.11 (1.80 to 2.48)
Diabetes		1.11 (1.02 to 1.20)	1.06 (0.97 to 1.15)
Hypertension		1.07 (0.98 to 1.16)	0.91 (0.83 to 0.99)
Heart failure		1.56 (1.44 to 1.68)	1.21 (1.11 to 1.32)
Cerebrovascular disease		1.44 (1.31 to 1.57)	1.22 (1.11 to 1.34)
Arrhythmia		1.67 (1.55 to 1.80)	1.29 (1.19 to 1.40)
Cancer		1.85 (1.65 to 2.07)	1.58 (1.40 to 1.77)
Chronic liver disease		1.43 (1.29 to 1.58)	1.59 (1.43 to 1.76)
Chronic obstructive pulmonary disease		1.53 (1.41 to 1.65)	1.20 (1.10 to 1.30)
Peripheral vascular disease		1.60 (1.48 to 1.72)	1.32 (1.22 to 1.44)
Valvular heart disease		1.48 (1.37 to 1.60)	1.20 (1.11 to 1.31)
History of kidney transplantation		0.77 (0.65 to 0.92)	0.98 (0.82 to 1.19)
History of ANVUGIB		0.79 (0.73 to 0.86)	0.76 (0.70 to 0.82)
Coronary artery disease*		1.24 (1.15 to 1.34)	
	Among men		0.87 (0.78 to 0.98)
	Among women		1.04 (0.92 to 1.18)

Abbreviations: ANVUGIB – acute nonvariceal upper gastrointestinal bleed; CAD – coronary artery disease.

* There was evidence for effect modification between sex and CAD in fully-adjusted models. Therefore, effect estimates are presented for sex within strata of CAD and for CAD within strata of sex. All estimates arose from models that were adjusted for calendar year, age, gender, race, dialysis vintage, modality, history of renal transplantation, prior ANVUGIB, hospitalized episodes, peptic ulcer related episodes, comorbidities (including diabetes, heart failure, coronary artery disease, chronic obstructive pulmonary disease, cerebral vascular disease, hypertension, arrhythmia, valvular heart disease, peripheral vascular disease, chronic liver disease and malignancy), and interaction between sex and coronary artery disease.

Only one interaction satisfied the validation criteria outlined in the Methods section (see Additional file 2): sex modified the association between coronary artery disease and 30-day mortality after ANVUGIB, where the odds of death were 13% lower in men with coronary artery disease than those without, whereas there was no association between coronary artery disease and death in women (Table 3).

## Discussion

We built a contemporary cohort using validated stringent algorithms and captured initial and repeat ANVUGIB episodes. Our sample size was uniquely and sufficiently large to empower us to investigate thoroughly numerous factors and interactions even under a very stringent type I error (0.01) and to split our data to avoid reporting false positive interactions. Furthermore, we tested the associations between dialysis specific parameters (such as dialysis modality and dialysis vintage) and ANVUGIB mortality for the first time. To our knowledge, this is the first population-based claims data analysis dedicated to studying risk factors for short-term mortality after ANVUGIB in patients on dialysis.

Cardiovascular disease, current smoking, and risk factors suggesting more disability had been reported to associate with a higher risk of UGIB among patients with ESRD [2]. In addition, we found several dialysis-specific parameters, such as using peritoneal dialysis rather than hemodialysis and longer dialysis vintage, to be associated with short-term mortality. Other predictors including older age and presence of most comorbid conditions were also associated with 30-day mortality, results not dissimilar from studies from non-ESRD populations. Furthermore, these factors are also risk factors for mortality in ESRD patients in general, even in the absence of ANVUGIB.

It was previously reported that most patients (79.7%) who experienced an ANVUGIB episode died of causes that were not directly bleeding-related. In a study of more than 10,000 patients in Hong Kong with peptic ulcer disease-related bleeding, Sung et al. showed that multi-organ system failure (23.9%), pulmonary conditions (23.5%) and terminal malignancy (33.78%) were the most common causes of death [3]. As a result, age, comorbidities and general health conditions were consistently found to be strong predictors of ANVUGIB mortality, whereas bleeding-specific parameters such as cause of bleeding, endoscopic findings, or treatments were not consistently associated with short-term outcome of ANVUGIB [6,13]. As expected, we showed that advanced age, hospitalized episodes, and most co-morbidities were independently associated with 30-day mortality after ANVUGIB among patients on dialysis, similar to findings from the general population, while bleeding from peptic ulcer disease rather than from any other cause did not significantly associate with short-term mortality after ANVUGIB. Although only 12.7% patients on dialysis had chronic liver disease, it was the most prominent predictor among all comorbidities in the full model (OR: 1.59; 99% CI: 1.43 to 1.76). In other studies of patients in the general population who experienced an upper gastrointestinal bleed, mortality following variceal hemorrhage was found to be higher than after ANVUGIB [6,10]. However, even for ANVUGIB, cirrhosis and liver failure were important comorbidities to predict mortality [5,6], which our study now confirms in a dialysis population. Similarly, patients with malignancy were more likely to die after UGIB both in the dialysis and the general population [4,5,14].

We found the association between CAD and mortality to be overall null, but modified by sex in that men, but not women, had a lower risk of mortality after a ANVUGIB episode (13% lower compared with otherwise similar men without CAD). Rockall et al. reported an unadjusted odds ratio for mortality of 4.3 (95% CI: 3.2-5.8) of ischemic heart disease in a prospective multicenter study of 4,185 cases of acute upper gastrointestinal bleeding [4]. By contrast, Klebl et al. found more cardiovascular disease (53.8% vs. 39.6%) among those who survived upper gastrointestinal bleeding than among those who died [15]. Several other studies reported only unspecified cardiovascular comorbidity as a risk factor for ANVUGIB related mortality [5,7,14,16]. Regarding aspirin use, two studies reported associations between use of aspirin and lower 30-day mortality [13,15], whereas others found no such association [5,7]. In reviewing the evidence, it appears that patients with ischemic heart disease represent a unique subgroup of ANVUGIB patients, perhaps with a different pathophysiology of bleeding, and further evaluation of ischemic heart disease as a risk factor for mortality after ANVUGIB is needed.

Our finding that patients undergoing peritoneal dialysis had higher short-term mortality after an ANVUGIB event compared with otherwise similar patients on hemodialysis is interesting. The association between dialysis modality and mortality has been widely studied, and it appears that older patients, especially women and those with diabetes, and those with longer vintage experienced better survival on hemodialysis [17-20]. We did not find any interactions among dialysis modality and these factors. While it is possible that peritoneal dialysis patients are more vulnerable when they experience an ANVUGIB, e.g. due to their altered gastrointestinal hemodynamics and physiology, it is also possible that patients undergoing hemodialysis receive more frequent medical surveillance, including monitoring of anemia, and ANVUGIB may thus be more likely to be detected earlier and treated more aggressively. Whether dialysis adequacy correlated with short-term outcome after ANVUGIB or not could not be answered in current study. It is possible that some patients undergoing peritoneal dialysis may achieve lower clearance and be more uremic, which may then contribute to worse bleeding, reduced hemostasis, and increased mortality risk.There was a graded increase of risk among patients with longer vintage. The association with vintage became significant mainly after adding age and prior ANVUGIB history into the model. The percentage of prior ANVUGIB increased with longer vintage (29.4%, 36.2% and 41.3% for vintage 1–3 years, 3–6 years and >6 years respectively), while age was negatively correlated with vintage (r=-0.19, p<0.001). Because prior ANVUGIB and younger age were associated with better outcomes, they confounded the association of vintage in the unadjusted model. Compared to those with dialysis vintage between 1 and 3 years, the odds ratios for 30-day mortality after ANVUGIB were 1.11 (99% CI: 1.01-1.21) and 1.37 (99% CI: 1.24-1.52) for those with dialysis vintage of 3–6 years and more than 6 years respectively. Longer duration of dialysis is associated with more severe dialysis or uremia related complications, such as cardiovascular calcification [21] or systemic amyloidosis [22-24]. These complications might weaken the capacity of patients on dialysis to pass through the stress of ANVUGIB. In addition, these complications may reduce the patient’s capability to achieve hemostasis once a bleeding occurs.

Prior history of ANVUGIB is another rarely mentioned factor for mortality after ANVUGIB, which is important especially for dialysis patients who are at particular risk of recurrence [1,25,26]. The longitudinal nature of the USRDS provided useful information on previous events, which could not be observed in other (prospective) cohorts with short inclusion and observation periods [4,5,7,15]. In a study of patients without ESRD, a history of bleeding ulcers was associated with bleeding continuation/re-bleeding within 30 days of the initial ANVUGIB episode, but not with 30-day mortality [13]. In our study, we considered re-bleeding within 30 days as being part of the same episode. We observed a 24% lower mortality for patients with a historical claims indicating prior ANVUGIB. However, this finding may reflect survivor bias, a special case of selection bias, or may be confounded by the duration of Medicare coverage prior to the index event. Patients who survived a previous episode of ANVUGIB may be more robust in ways that is beyond what can be observed in a claims dataset. In addition, such episodes may have been milder bleeds, and be followed by other milder bleeding episodes. Further, patients who survived previous bleeds may have received adequate intervention, medical treatment, or heightened surveillance, leading to milder subsequent bleeding episodes. Unfortunately, the USRDS does not contain information on medications including eradication of *H. pylori* or long-term proton pump inhibitor therapy. Our findings also suggest that clinicians need to be particularly vigilant when patients on dialysis experience an initial episode of ANVUGIB and be aware of the heightened mortality risk in patients experiencing an initial bleed.

In the dialysis population, black individuals experience better long-term survival then whites [27,28]. Compared to white patients in our cohort, black patients also had significantly lower risk for 30-day mortality after ANVUIGB (OR: 0.80; 99% CI: 0.73-0.87) after multivariable adjustments. In the general population, race has been rarely linked to poor outcome after UGIB. Among elderly patients admitted with GIB using data from Medicare Provider Analysis and Review (MedPAR) File and the VA Patient Treatment File (PTF), 30-day mortality for black patients was 6.8%, compared with 7.8% for white patients (p<0.05). The difference remained significant (1.4%; p<0.05) after adjustment for baseline comorbid conditions, socioeconomic variables, and hospital effects [29]. The racial disparities in short-term outcome after ANVUGIB might result from biologically racial differences of gastrointestinal pathology or stress reactions which deserve further study.

Despite those strengths of this study, we need to acknowledge certain limitations. The most important limitation stems from the use of administrative billing claims to identify the event of interest. However, the algorithm we used to identify ANVUGIB episodes had been validated by Cooper et al. [30] and modified by Targowinik et al. [8]. In addition, we applied a rather stringent criterion to ascertain ANVUGIB episodes to increase the specificity of the algorithm. Nevertheless, severe episodes from which patients died before ANVUGIB could be diagnosed or coded may not have been captured by our data.

Another limitation is that we did not have information on biometric measurements, laboratory results and endoscopic findings. Hence, the *c*-statistic of the fully-adjusted model was only 0.68. However, in daily practice, such clinical characteristics may remind clinicians of higher risk of short-term mortality from ANVUGIB in patients on dialysis, even before laboratory data or endoscopy findings are available.

In order to ensure correct ascertainments of co-morbidities status from claims, we further restricted our cohort to only those patients under Medicare as primary payer for at least one year before the ANVUGIB dates. Thus, the median dialysis vintage of this cohort (3.8 years) was longer than that of the general prevalent dialysis population and we do not know whether our results generalize to those patients dialyzed for less than one year or to those who have primary payers other than Medicare.

## Conclusions

In conclusion, patients on dialysis represent a unique subgroup of patients with ANVUGIB exhibiting high risk for mortality and distinct risk factors. Patients with older age, longer dialysis vintage, several co-morbidities, no previous history of ANVUGIB, and those receiving peritoneal dialysis were at higher risk for 30-mortality after ANVUGIB.

### Ethical approval

The Stanford University School of Medicine Institutional Review Board approved of this study. The data reported here have been supplied by the USRDS. The interpretation and reporting of these data are the responsibility of the authors and in no way should be seen as an official policy or interpretation of the US government. This work was conducted under a data use agreement between W.C.W. and the National Institute of Diabetes, Digestive and Kidney Diseases (NIDDK). This manuscript was reviewed by NIDDK and approved for submission.

## Abbreviations

AIC: Akaike information criterion; ANVUGIB: Acute nonvariceal upper gastrointestinal bleeding; ESRD: End-stage renal disease; GEE: Generalized estimating equations; USRDS: United States Renal Data System

## Competing interests

W.C.W. has served as a scientific advisor or consultant to Affymax, Amgen, Bayer, Fibrogen, and GlaxoSmithKline. He has received unrestricted research support from Fibrogen. G.M.C. serves on the Board of Directors of Satellite Healthcare, Inc., and on the Scientific Advisory Board of DaVita Clinical Research and has received research support and served as an advisor to Amgen. No other author has reported any competing interest.

## Authors’ contributions

All authors were involved in the study design and concept, interpretation of results, editing and final approval of manuscript. All authors had full access to all of the data (including statistical reports and tables) in the study. All authors take responsibility for the integrity of the data and the accuracy of the data analysis. All authors read and approved the final manuscript.

## Pre-publication history

The pre-publication history for this paper can be accessed here:

http://www.biomedcentral.com/1471-2369/14/97/prepub

## Supplementary Material

Additional file 1 **Appendix 1.** Algorithms to identify ANVUGIB from Medicare claims. Click here for file

Additional file 2 **Appendix 2.** Akaike information criterion (AIC) of main effect models or main effect plus interaction term models fit to the derivation and validation samples. Click here for file
