# Supplementary material for: Risk factors of short-term mortality after acute nonvariceal upper gastrointestinal bleeding in patients on dialysis: a population-based study
Source: BMC Nephrol. 2013 Apr 26;14:97. doi: 10.1186/1471-2369-14-97 (PMC3639820; doi:10.1186/1471-2369-14-97)
Supplement: Additional file 1 — Appendix 1. Algorithms to identify ANVUGIB from Medicare claims. [file 1471-2369-14-97-S1.pdf]

Appendix 1: Algorithms to identify ANVUGIB from Medicare claims

1. Diagnosis in group A
2. Exclude diagnosis in group B (coding of Esophageal variceal bleeding, Gastric variceal bleeding)

Plus one of the following condition:

1. Inpatient setting
2. Criteria at least twice within 7 days
3. Criteria + gastroscopy on the same day

Group A: specific diagnosis for NVUGIB	
ICD-9 code	Diagnosis
530.21	Ulcer of esophagus with bleeding
530.7	Gastroesophageal laceration-hemorrhage syndrome (Mallory-Weiss syndrome)
530.82	Esophageal hemorrhage
531.0X	Gastric ulcer, Acute with hemorrhage
531.2X	Gastric ulcer, Acute with hemorrhage and perforation
531.4X	Gastric ulcer, Chronic or unspecified with hemorrhage
531.6X	Gastric ulcer, Chronic or unspecified with hemorrhage and perforation
532.0X	Duodenal ulcer, Acute with hemorrhage
532.2X	Duodenal ulcer, Acute with hemorrhage and perforation
532.4X	Duodenal ulcer, Chronic or unspecified with hemorrhage
532.6X	Duodenal ulcer, Chronic or unspecified with hemorrhage and perforation
533.0X	Peptic ulcer, Acute with hemorrhage
533.2X	Peptic ulcer, Acute with hemorrhage and perforation
533.4X	Peptic ulcer, Chronic or unspecified with hemorrhage
533.6X	Peptic ulcer, Chronic or unspecified with hemorrhage and perforation
534.0X	Gastrojejunal ulcer, Acute with hemorrhage
534.2X	Gastrojejunal ulcer, Acute with hemorrhage and perforation
534.4X	Gastrojejunal ulcer, Chronic or unspecified with hemorrhage
534.6X	Gastrojejunal ulcer, Chronic or unspecified with hemorrhage and perforation
535.X1	Gastritis and duodenitis with hemorrhage
537.83	Angiodysplasia of stomach and duodenum with hemorrhage
537.84	Dieulafoy lesion (hemorrhagic) of stomach and duodenum
578.0	Hematemesis
Group B: diagnosis specific for esophageal variceal bleeding	

456.0	Esophageal varices with bleeding
456.2	Esophageal varices in diseases classified elsewhere with bleeding

We searched for all institutional claims and physician/supplier claims. For institutional claims, those sources of the bill valued “inpatient” or “PMMIS inpat stay” were labeled as “inpatient”. For physician/supplier claims, “inpatient” referred to those places of service valued “inpatient hospital”. Otherwise, the claims were labeled as “outpatient”. Both institutional claims and physician/supplier claims for the endoscopic procedure codes.
