# Supplementary material for: Risk factors of short-term mortality after acute nonvariceal upper gastrointestinal bleeding in patients on dialysis: a population-based study
Source: BMC Nephrol. 2013 Apr 26;14:97. doi: 10.1186/1471-2369-14-97 (PMC3639820; doi:10.1186/1471-2369-14-97)
Supplement: Additional file 2 — Appendix 2. Akaike information criterion (AIC) of main effect models or main effect plus interaction term models fit to the derivation and validation samples. [file 1471-2369-14-97-S2.pdf]

Appendix 2: Akaike information criterion (AIC) of main effect models or main effect plus interaction term models fit to the derivation and validation samples.

Significant interaction terms in development sample		AIC_d	AIC_v
Main effect model		19534.9	13128.6
Age interacted with:	Cerebral vascular disease	19531.1	13131.6
	Valvular heart disease	19524.8	13132.2
	Peripheral vascular disease	19526.9	13131.5
	Chronic liver disease	19526.2	13136.7
	Prior ANVUGIB history	19535.5	13133.5
	Cancer	19530.9	13139.8
Sex interacted with:	Hypertension	19530.1	13134.2
	Coronary artery disease	19531.8	13127.5*
Race interacted with:	Prior ANVUIGB history	19534.7	13135.5
	Peripheral vascular disease	19533.5	13137.5
	Hospitalizied episodes	19534.0	13134.8

*AIC_d: AIC of derivation sample.

**AIC_v: AIC computed in the validation sample using parameter estimates from the derivation sample.

Note: There were no significant interactions among modality and other covariates in the derivation sample.
